# Targeted Accumulation of Macrophages Induced by Microbeam Irradiation in a Tissue-Dependent Manner

**DOI:** 10.3390/biomedicines10040735

**Published:** 2022-03-22

**Authors:** Verdiana Trappetti, Jennifer Fazzari, Cristian Fernandez-Palomo, Lloyd Smyth, Marine Potez, Nahoko Shintani, Bettina de Breuyn Dietler, Olga A. Martin, Valentin Djonov

**Affiliations:** 1 Institute of Anatomy, University of Bern, Baltzerstarsse 2, 3012 Bern, Switzerland; verdiana.trappetti@unibe.ch (V.T.); jennifer.fazzari@unibe.ch (J.F.); cristian.fernandez@unibe.ch (C.F.-P.); marine.potez@gmail.com (M.P.); nahoko.shintani@unibe.ch (N.S.); bettina.debreuyn@unibe.ch (B.d.B.D.); olga.martin@unibe.ch (O.A.M.); 2 Department of Obstetrics and Gynaecology, Royal Women’s Hospital, University of Melbourne, Melbourne, VIC 3052, Australia; lloyd.smyth@icon.team; 3 Department of Immunology, H. Lee Moffitt Cancer Center and Research Institute, 12902 USF Magnolia Drive, Tampa, FL 33612, USA; 4 Division of Radiation Oncology, Peter MacCallum Cancer Centre, 305 Grattan St., Melbourne, VIC 3000, Australia; 5 Department of Oncology, University of Melbourne, Parkville, VIC 3010, Australia

**Keywords:** microbeam radiotherapy, DNA damage, macrophages, infiltration

## Abstract

Radiation therapy (RT) is a vital component of multimodal cancer treatment, and its immunomodulatory effects are a major focus of current therapeutic strategies. Macrophages are some of the first cells recruited to sites of radiation-induced injury where they can aid in tissue repair, propagate radiation-induced fibrogenesis and influence tumour dynamics. Microbeam radiation therapy (MRT) is a unique, spatially fractionated radiation modality that has demonstrated exceptional tumour control and reduction in normal tissue toxicity, including fibrosis. We conducted a morphological analysis of MRT-irradiated normal liver, lung and skin tissues as well as lung and melanoma tumours. MRT induced distinct patterns of DNA damage, reflecting the geometry of the microbeam array. Macrophages infiltrated these regions of peak dose deposition at variable timepoints post-irradiation depending on the tissue type. In normal liver and lung tissue, macrophages clearly demarcated the beam path by 48 h and 7 days post-irradiation, respectively. This was not reflected, however, in normal skin tissue, despite clear DNA damage marking the beam path. Persistent DNA damage was observed in MRT-irradiated lung carcinoma, with an accompanying geometry-specific influx of mixed M1/M2-like macrophage populations. These data indicate the unique potential of MRT as a tool to induce a remarkable accumulation of macrophages in an organ/tissue-specific manner. Further characterization of these macrophage populations is warranted to identify their organ-specific roles in normal tissue sparing and anti-tumour responses.

## 1. Introduction

Despite recent advances in cancer therapy, the prognosis remains poor in many clinical cases. Radiotherapy (RT) has been the subject of technological innovation over the years as an essential component of treatment protocols for over 50% of all cancer patients [1]. Despite recent advances, the capabilities of RT in tumour control are still limited by normal tissue thresholds for radiation toxicity. In some cases, this prevents the appropriate dose escalation necessary to overcome radioresistance and limits its use for treating malignancies in particularly sensitive organs. Advances in the field of RT are therefore aimed at innovating the current clinical methodology in order to improve therapeutic outcomes.

Synchrotron microbeam radiation therapy (MRT) is a novel, pre-clinical RT modality that combines micrometric spatial fractionation and FLASH dose-rates to maximize the therapeutic ratio. Synchrotron-generated X-rays pass through a collimator to produce micro-planar beams of radiation at very high doses (up to thousands of Gray (Gy)) and dose-rates (up to 16,000 Gy/s). Microbeam widths range from 20 to 100 µm, while the spacing between them ranges from 50 to 500 µm. MRT provides enhanced tumour control relative to conventional RT, as the spatial fractionation and ultra-high dose rate allows for substantial dose escalation with significantly reduced collateral damage to normal tissues within the irradiation field [2,3]. The mechanisms behind the efficacy of MRT have not been fully elucidated, but some data have implicated macrophages as an important player in its therapeutic response [4,5,6,7].

Macrophages have an important pathophysiological role in normal tissue responses after radiation injury, a type of sterile injury. Studies have shown that ionizing radiation induces immunomodulatory effects [8] which can be either tolerogenic or immunogenic [9,10], depending on the distinct downstream signalling mechanisms that influence tissue repair [11,12]. Such responses involve a complex cascade of events that depend on a variety of factors, including the dose and fractionation, target site and volume of tissue irradiated. The type of cell death mechanisms induced by RT also influences the nature of the immune response [13]. The production of damage-associated molecular patterns (DAMPs) from irradiated cells elicits innate immune activation [12], with macrophages being one of the first immune cells to respond to sites of radiation-induced injury [14,15,16,17].

Normal tissue responses are particularly important to understand in order to avoid treatment-induced organ dysfunction. For example, the lung and liver are two of the most sensitive organs in terms of long-terms effects (e.g., radiation-induced fibrosis) and are often associated with collateral radiation toxicity in clinical RT regimens. Following conventional radiation of the lung, dynamic changes in immune cell populations at the site of irradiation are observed both in the parenchyma and alveoli [14]. Similarly, the liver is susceptible to radiation-induced disease and long-term toxicities [18]. Furthermore, all external irradiation regimens involve skin penetration and, therefore, the risk of cutaneous radiation injury. Overall, the radiation dose prescription is dictated by the radiation toxicity thresholds of such normal tissues, in some cases limiting its use entirely for primary malignancies that involve significant lung and liver exposure. It is therefore important when employing new RT modalities, such as MRT, to understand the influence of the modality on macrophage dynamics in normal tissues. It has been shown that exposure of the skin on the murine hind limb to a short pulse of MRT or broad beam (BB) prompted an increased frequency of macrophages in both the irradiated skin and out-of-field tissues [19]. An increase in tumour necrosis factor (*Tnf*) expression, a gene associated with inflammation, including promotion of macrophage activation, further underlines the role of macrophages in the normal tissue response to MRT [7].

It is now known that local immune responses also play an important role in MRT tumour control efficacy (reviewed in [20]). In tumours, endogenous cytokine and chemokine release from the tumour microenvironment attracts immune cells to the tumour, initiating a cascade of immunostimulatory responses [21]. Ionizing radiation attracts macrophages to irradiated tumours [22,23,24] in response to the radiation-mediated release of pro-inflammatory cytokines [25]. Tumour-associated macrophages (TAMs) form a substantial proportion of the infiltrating leucocytes in lung tumours and are an emerging target for lung cancer therapies [26]. TAMs in lung tumours can exhibit two opposite phenotypes: M1-like or M2-like, with the first displaying anti-inflammatory properties and the latter promoting tumour growth, invasion, and neo-angiogenesis [26,27].

Previous data have shown that MRT irradiation of a B16F10 melanoma resulted in an increase in chemokines associated with the migration and influx of inflammatory monocytes. These included monocyte chemoattractant protein (MCP)-1, macrophage inflammatory protein (MIP)-1α and MIP-1β, C-C motif ligand 5 (CCL5), as well as interleukin (IL)12p40 [4]. This was associated with an increased infiltration of CD68+ cells, especially at 9 days post-MRT [4]. In a subsequent study, complete remission of this same tumour type following MRT was characterized by an agglomeration of macrophages containing phagocytized melanin granules (melanophages) 18 months after treatment [5]. Furthermore, Yang and colleagues [6] studied the effects of MRT in comparison to homogenous irradiation (BB) on the macrophage content in murine mammary carcinomas. A general decrease in tumour-associated-macrophages (TAMs) was observed, as reflected by both a low percentage of M1-like (Ly6C^hi^) and M2-like (Ly6C^low^) TAMs following MRT, while the BB group showed a significant increase relative to the un-irradiated controls [3,6].

The role of macrophages following radiation, therefore, dictates important radiation-induced outcomes for both normal tissue tolerance and tumour responses to RT. In order to further investigate the macrophage response following MRT, we selected a panel of normal tissues and tumours given their relevance to our experimental models and with the potential for future clinical applications. MRT-irradiated lung carcinomas from a previous survival study [28] were examined in combination with collaterally irradiated normal liver and healthy lung tissues. In addition, MRT-irradiated normal mouse ear pinnae [29] and mouse melanomas were also examined. The investigated sites are distinctive in their composition, cellular turnover and susceptibility to radiation (i.e., the lung and liver are two of the most sensitive organs to long-term radiotoxicity, while melanoma and lung carcinoma have unmet clinical needs often attributed to radioresistance). Furthermore, each have distinct roles associated with resident or tumour-associated macrophage populations, providing a suite of clinically relevant tissues to examine macrophage behaviour following MRT.

## 2. Materials and Methods

### 2.1. Animals, Cell Lines and Irradiations

Protocols related to animals, cell lines, tumour inoculation, irradiation set-up, dosimetry and post-mortem tissue fixation for the lung [28] and melanoma/skin studies [4,29] have been described previously. All samples presented in this study are collected from female C57BL/6J mice purchased from either Charles River laboratories (Les Oncins, France) for those experiments performed at the European Synchrotron Radiation Facility or in Bern, or from the Animal Resources Centre (Canning Vale, WA, Australia) for experiments performed at the Australian Synchrotron (Clayton, VIC, Australia). All experiments received ethical approval from the appropriate authorities in the country in which they were performed. They include the following approvals: the Veterinary Office of Bern (Bern, Switzerland), approval code: BE61/15 and approval date: 2015; the European Synchrotron Radiation Facility (ESRF, Grenoble, France) Internal Evaluation Committee for Animal Welfare and Rights, approval code: 14_ethax22 and approval date: 2014; the Australian Synchrotron Animal Ethics Committee, approval code: AS2019_003 and approval date: 2019; and the Australian Synchrotron Animal Ethics Committee, approval code: AS2019_007 and approval date: 2019.

#### 2.1.1. MRT Irradiation of Lung Carcinoma and Collateral Irradiation of the Liver

Orthotopic Lewis Lung Carcinoma (LLC1)-bearing mice were irradiated with 2 cross-fired MRT arrays (50 µm width, 400 µm spacing) with peak entrance doses of 400 Gy as previously described [28].

Given the location of the carcinoma in the lower right lung, the medial lobe of the liver was also collaterally irradiated. Therefore, as an organ at risk, the liver was collected for the examination of collateral effects of MRT on this organ. The cellular density and regeneration capacity of the liver in conjunction with radiation-induced toxicities made it an organ of interest to assess preliminary responses to MRT.

#### 2.1.2. MRT Irradiation of Normal Mouse Ear Pinnae and Melanoma

The normal ear pinnae of B16F10 melanoma-bearing mice were irradiated as previously described [4,29] with a single MRT array (50 µm width, 200 µm spacing) with an 800 Gy peak dose for the normal ear and a 400 Gy peak dose for melanoma.

A summary of all tissue types investigated, corresponding irradiation parameters, markers employed and time points is reported in Table 1.

### 2.2. Immunohistochemistry

Immunohistochemistry was performed on 5-μm-thick paraffin sections of the lungs (normal and carcinoma-bearing) and normal ears, 3-μm-thick paraffin sections of the livers and 12-μm-thick frozen sections of the melanoma-bearing ears. At least 5 animals were included per treatment group. Tissues were embedded and processed for histology. Serial sectioning was performed and representative images are shown. The following primary antibodies were employed: a rabbit polyclonal antibody against pan-macrophage biomarker CD68 (1:500, ab125212, Abcam plc, Cambridge, UK) for lung carcinoma and normal ear sections; a rabbit monoclonal antibody against CD68 (EPR23917-164) (1:1600, ab283654, Abcam plc) for normal lung sections; a rat monoclonal antibody against the pan-macrophage marker CD68 (FA-11) (1:2000, MCA1957, Bio-Rad Laboratories Inc., Hercules, CA, USA) for melanoma-bearing ears; goat polyclonal antibodies against the surface protein CD206 (1:50, AF2535) and the transcription factor GATA-6 (1:200, AF1700, R&D Systems, Minneapolis, MN, USA); rabbit polyclonal antibodies against the surface protein CD11b (1:3000, NB110-89474, Novus Biologicals, Centennial, CO, USA) and a DNA damage marker γH2A.X (1:200, ab11174, Abcam plc) and rat monoclonal antibodies against pan-macrophage biomarker F4/80 [CI:A3-1] (1:100, ab6640, Abcam plc), the surface protein Dectin-1 [2A11] (1:100, MA5-16477 Invitrogen) and the surface protein Ly6C [ER-MP20] (1:200, MA1-81899, Invitrogen, Carlsbad, CA, USA). Deparaffinised sections underwent antigen retrieval (sodium citrate buffer, pH 6 for γH2A.X; Tris-EDTA buffer, pH 9 for CD68, CD11b, GATA-6, Ly-6C, Dectin-1 and CD206; proteinase K solution for F4/80). After a blocking step, the sections were incubated with the primary antibody. Endogenous peroxidase activity was blocked with 0.6% hydrogen peroxide (3% for F4/80). To detect primary antibodies, the following secondary antibodies (all from Vector Laboratories, Burlingame, CA, USA) were employed: ImmPRESS goat anti-rabbit IgG (1:1, MP-7451) for CD68, CD11b and γH2A.X; ImmPRESS horse anti-goat IgG (1:1, MP-7405) for GATA-6 and CD206 and biotinylated goat anti-rat (1:200, 112-065-062, Jackson Immuno Research, Ely, UK) for F4/80. The sections were stained with NovaRED (SK-4800, Vector Laboratories), and cell nuclei were counterstained with haematoxylin. Stained sections were evaluated and photographed in an IMAGER.M2 light microscope (Carl Zeiss Microscopy GmbH, Jena, Germany).

For double immunofluorescence staining, after incubation with the primary antibodies CD206 and CD68 or Ly6C and CD68, slices were incubated with donkey anti-goat Alexa Flour 488 (A-11055) and donkey anti-rabbit Alexa Fluor 568 (A10042) or donkey anti-rat Alexa Flour 488 (A-21208) and donkey anti-rabbit Alexa Fluor 568 (A10042) (1:200, ThermoFisher Scientific, Fremont, CA, USA), respectively, together with 4′,6-diamidino-2-phenylindole (DAPI, 1:1000). For triple immunofluorescence staining, after incubation with the primary antibodies CD206, Dectin-1 and CD68, slices were incubated with donkey anti-goat Alexa Flour 488 (A-11055), donkey anti-rabbit Alexa Fluor 568 (A10042) and donkey anti-rabbit Alexa Fluor 647 (A-31573) (1:200, ThermoFisher Scientific, Fremont, CA, USA) and DAPI (1:1000). A Leica SP8 (Leica Camera AG, Wetzlar, Germany) or a Zeiss LSM 880 (Carl Zeiss Microscopy GmbH, Jena, Germany) confocal microscope were employed to image the sections.

## 3. Results

### 3.1. Normal Tissues

#### 3.1.1. Liver

**Figure 1:**
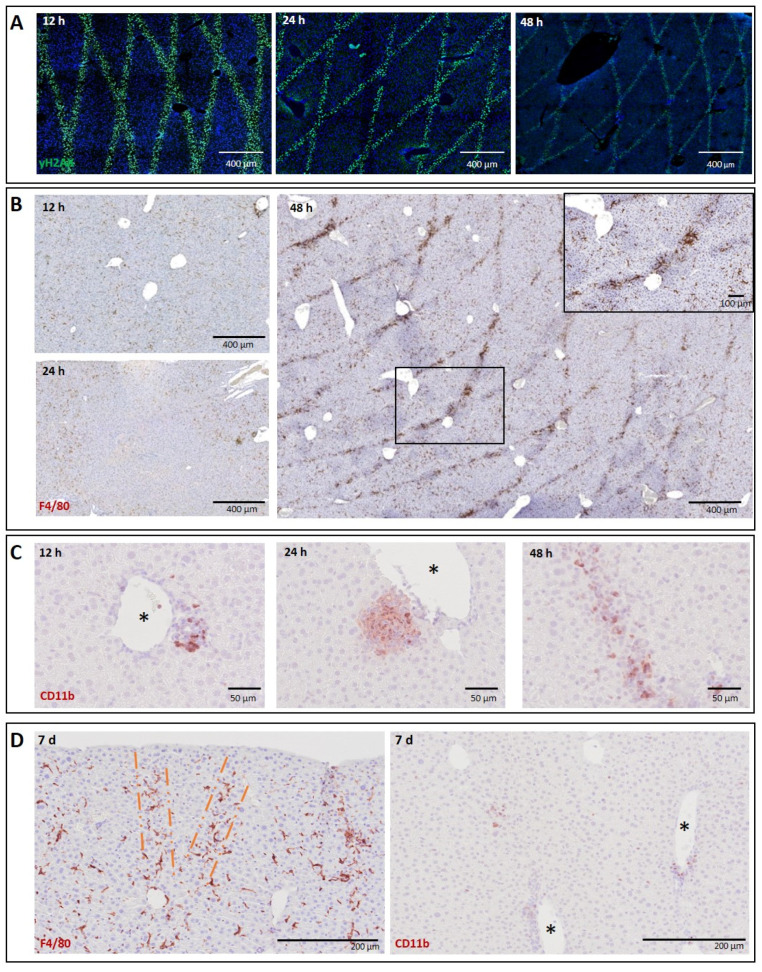
Time course of macrophage infiltration into MRT beam path in the irradiated liver. (**A**) γH2AX staining (green fluorescent cells over DAPI) in normal liver tissue at 12, 24 and 48 h post-MRT irradiation. (**B**) F4/80 staining (DAB) in normal liver tissue at 12, 24 and 48 h post-MRT irradiation. Highest concentration in beam path by 48 h with higher magnification of F4/80-positive macrophages in cross-fired region shown as insert. (**C**) CD11b+ cell populations (NovaRED) appearing around central veins at 12 and 24 h post-MRT and migrating to the beam path by 48 h. (**D**) At 7 days post-irradiation, macrophage organization persists along the beam, while CD11b staining significantly diminishes by this time. Orange dashed lines indicate clear macrophage “stripes”. * Asterisks indicate central veins.

Radiation tracks corresponding to the MRT geometry were clearly demarcated by the DNA damage biomarker, γH2AX [30] in examined liver tissue at 12, 24 and 48 h post-irradiation (Figure 1A). H2AX is a histone variant which is rapidly phosphorylated following the induction of DNA double-strand breaks, forming γH2AX foci at these damaged sites [31]. Macrophages are the most abundant immune population in this organ [32], with Kupffer cells comprising the vast majority of the liver resident macrophage population. By 48 h post-irradiation, a marked organization of macrophages along the radiation paths was observed; immunostaining for the pan-macrophage biomarker F4/80 revealed clear demarcation of the microbeam cross-fired geometry with a higher density of macrophages located at the points of highest dose deposition in the cross-fired regions (Figure 1B). Furthermore, CD11b+ cells, attributable to monocytic derived macrophage populations, appeared to accumulate near central veins at 12 h post-MRT and expanded along the beam path by 48 h (Figure 1C). Macrophage populations persisted in the beam path for extended periods of time. Demarcation of MRT geometry by F4/80+ macrophage populations was still visible, albeit with more dispersed organization, at 7 days post-irradiation, and CD11b+ populations were greatly reduced at this time point (Figure 1D).

Furthermore, macrophages localizing to the beam path were negative for GATA 6 (Supplementary Figure S1), a biomarker of peritoneal macrophages [33] known to respond to sterile injury of the liver [34].

#### 3.1.2. Lung

**Figure 2:**
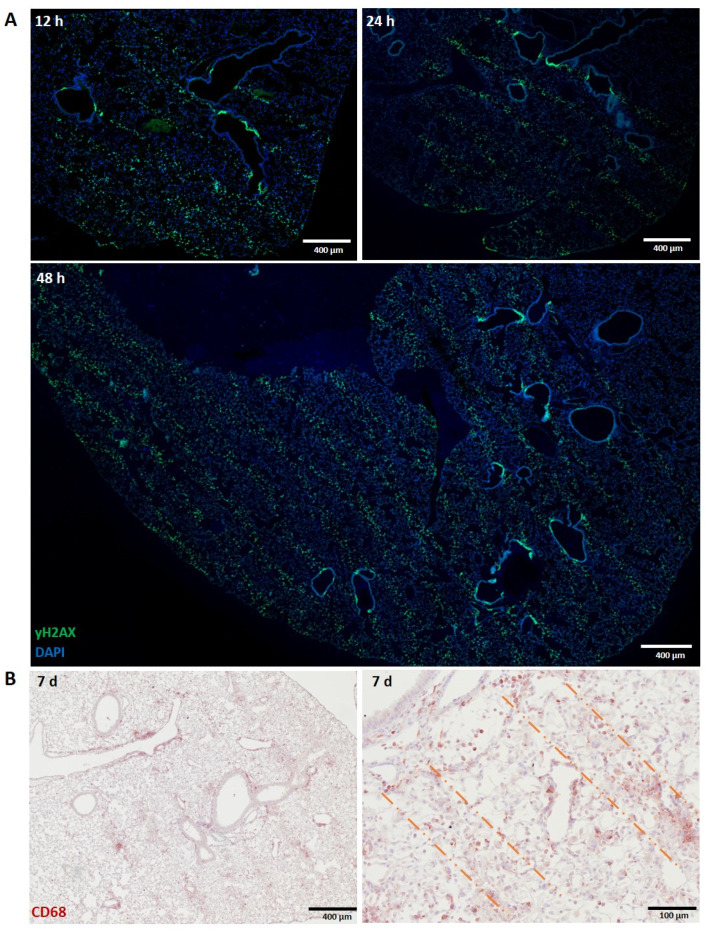
Macrophage distribution in normal lungs. (**A**) γH2AX staining at 12, 24 and 48 h post-MRT irradiation. (**B**) CD68 staining (NovaRED) in normal lung tissue at 7 days post-MRT irradiation, which is the earliest time point in which we could observed the patterned accumulation of macrophages. Dashed lines indicate clear macrophage “stripes”. Images at two different magnifications are shown.

The presence of macrophages was investigated in lung tissue harvested from healthy mice at early time points (12, 24 and 48 h) post-irradiation and at later time points in non-malignant lung tissue of MRT-irradiated lung carcinoma-bearing mice. The specific accumulation of alveolar macrophages along the microbeam path was more difficult to recognize in the normal lung due to its lower tissue density. Nevertheless, the same MRT geometry as described above for liver and shown previously [28] was still recognizable by both the DNA damage biomarker γH2AX at 48 h post-irradiation (Figure 2A) and the pan-macrophage biomarker, CD68, at 7 days post-MRT in irradiated lung carcinoma (Figure 2B). In contrast to the liver, normal lung tissue did not show this same specific, localized infiltration (data not shown) at 12, 24, 48 and 72 h post-MRT.

#### 3.1.3. Skin (Ear)

**Figure 3:**
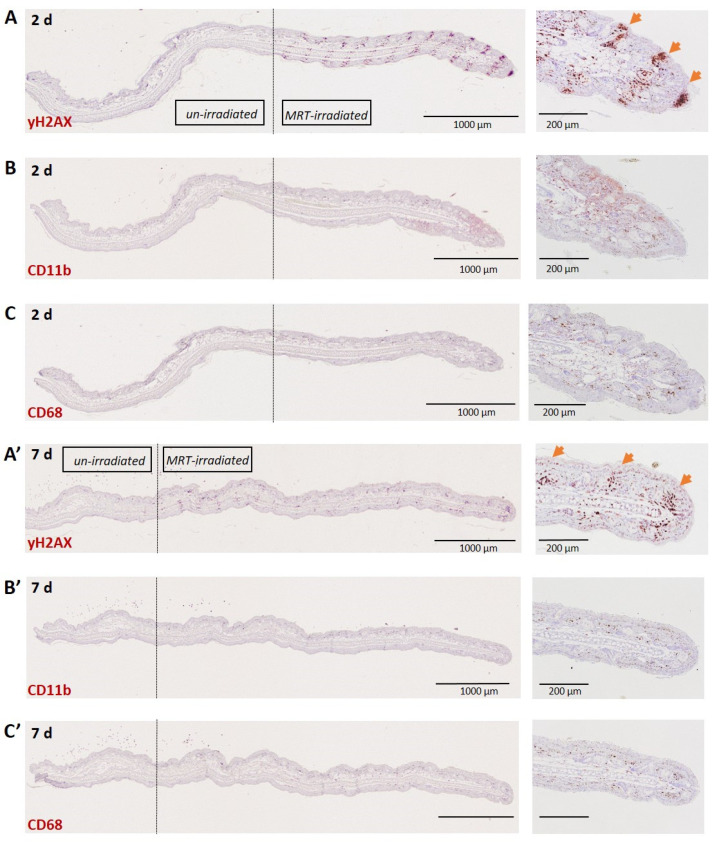
DNA damage and associated macrophage response following 800 Gy, 2 days (**A**–**C**) and 7 (**A’**–**C’**) days following MRT irradiation of the normal mouse ear. γH2AX immunostaining (**A**,**A’**) (NovaRED) depicting MRT beam paths (indicated by arrows). CD11b immunostaining (**B**,**B’**) (NovaRED). CD68 immunostaining (**C**,**C’**) (NovaRED).

The macrophage response following MRT was also examined in the normal skin of the mouse ear pinnae. DNA damage clearly identified the beam path at 2 (Figure 3A) and also 7 (Figure 3A’) days post-irradiation. Macrophages, however, did not localize to the beam path at these time points when examining both CD11b+ (Figure 3B,B’) and CD68+ (Figure 3C,C’) populations. Although there was an absence of beam-path-specific macrophage accumulation in the skin at these time points, a higher density of CD11b+ and CD68+ cells can be observed in the MRT-irradiated field towards the tip of the ear relative to the out-of-field segment towards the base of the ear.

### 3.2. Tumours

#### 3.2.1. Lung Carcinoma

**Figure 4:**
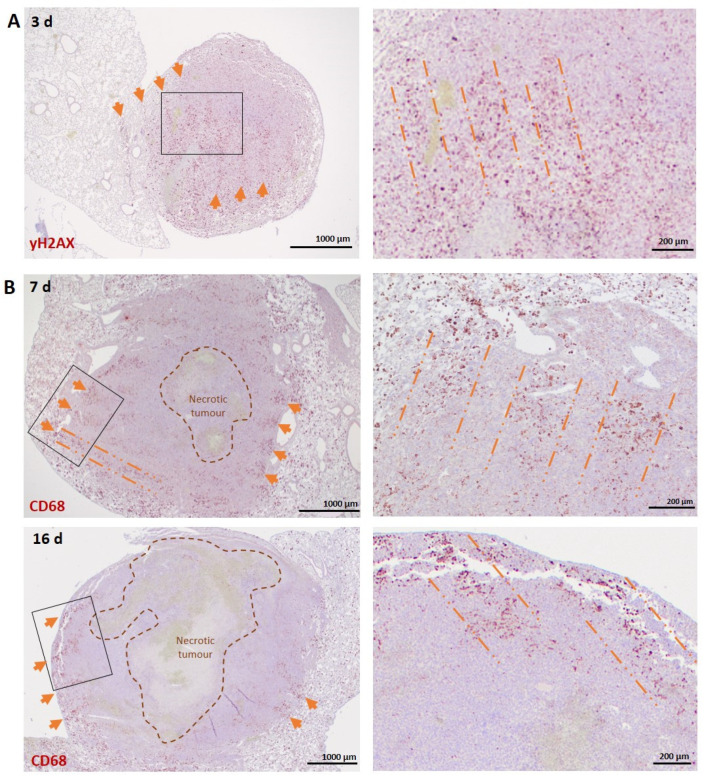
Macrophage staining reveals patterned infiltration in the periphery of lung carcinoma. (**A**) γH2AX staining (NovaRED) in LLC1 mouse carcinoma at 3 days post-MRT reveals persistent DNA damage and the adopted cross-firing geometry. Orange arrows and dashed lines indicate the microbeam paths. (**B**) CD68 staining (NovaRED) in LLC1 lung carcinoma at 7 and 16 days post-MRT irradiation. Orange arrows indicate the accumulated TAMs corresponding to microbeam geometry. Orange, dashed lines indicate a clear macrophage infiltration in the microbeam path. Images on the right panels are zoomed-in views of the regions indicated with a rectangle in the left panel.

Anti-γH2AX immunostaining at 3 days post-irradiation revealed a geometric pattern similar to that described above in the normal lung (Figure 4A). Interestingly, immunostaining with the anti-CD68 antibody revealed that the infiltration of TAMs followed the microbeam paths in mouse LLC1 carcinomas at 7 and 16 days post-irradiation, predominantly in the tumour periphery (Figure 4B). This is in contrast to non-irradiated tumours, in which macrophages are restricted to the tumour periphery with limited infiltration (Supplementary Figure S2).

**Figure 5:**
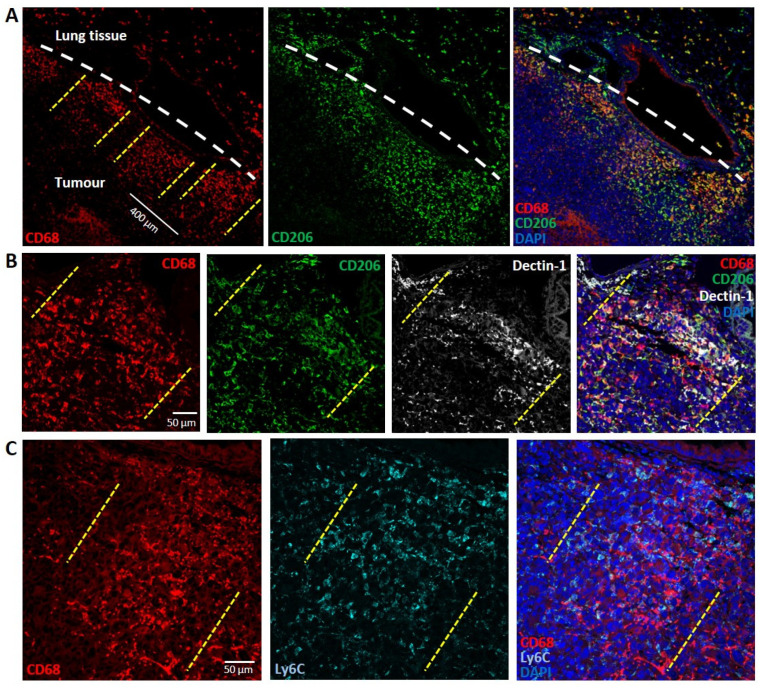
TAMs along the beam paths show a mixed M2-like/M1-like phenotype and expression of phagocytosis markers. (**A**) Double staining for CD68 (red) and CD206 (green) plus DAPI (blue) shows at 7 days post-MRT that many of the underlined TAMs are positive for both markers, indicating their inclination towards an M2-like phenotype. Dashed white lines demarcate the border between the tumour (lower part) and the normal tissue (upper part). (**B**) Triple staining for CD68 (red), CD206 (green) and Dectin-1 (grey) shows an abundant triple positive TAM population along the beam paths, indicating their inclination towards a phagocytic phenotype. (**C**) Double staining for CD68 (red) and Ly6C (cyan) reveals a partial M1-like TAM population and, at the same time, reveals cells that are not double positive, indicating the presence of recruited monocytes. Dashed yellow lines indicate a macrophage cluster along a microbeam path.

To further investigate the phenotype of the observed TAMs, double fluorescent staining with anti-CD68, a pan-macrophage biomarker and anti-CD206, a biomarker for M2-like TAMs, was performed on carcinoma sections at 7 days post-MRT. The CD68 signal confirmed the pattern previously observed with the chromogen staining, whilst CD206 did not show an exclusive co-localization with the CD68+ populations but was also abundant in the tumour periphery (Figure 5A). The markers CD206 and Dectin-1, although classically associated with the M2 phenotype, are expressed by phagocytic macrophages [35,36]. Triple positivity for CD68, CD206 and Dectin-1 identified a macrophage sub-population among the clustered macrophages in the microbeam areas, which show an active phagocytic response (Figure 5B). On the other hand, the co-localization of CD68 and Ly6C indicate a monocytic and potential M1-like macrophage component [37] mixed with the prominent M2-like populations. Cells positive for Ly6C but not CD68 in these areas may indicate active monocytic recruitment (Figure 5C).

#### 3.2.2. Melanoma

By 2 h post-irradiation, MRT induced distinct DNA damage patterns corresponding to the MRT geometry (Supplementary Figure S3A). Pan-macrophage staining with CD68 revealed peritumoural accumulation of macrophages at 2 days post-irradiation with an influx into the tumour at 7 days post-irradiation (Supplementary Figure S3B), confirming what was previously described at 9 days post-MRT [4]. This increase was also observed with CD206+ macrophages, although it is clear that the intra-tumoural presence of CD68+ cells is more consistent than the CD206+ cells, suggesting that a large part of the infiltrating macrophages might not be oriented towards an M2-like phenotype but might eventually be M1-like. (Supplementary Figure S3C). However, a clear localization of intra-tumoural macrophages within the microbeam path was not observed.

## 4. Discussion

Exploiting the unique immunomodulatory cascade following RT is a point of great interest for adjuvant cancer treatment strategies. Based on previous studies investigating the outcome of tumour-bearing animals treated with MRT, macrophages were implicated as early responders to MRT-induced DNA damage in normal and tumour tissues [6,38,39]. Activated macrophages mobilised towards the site of injury and had a predominant phagocytic action against apoptotic cells in acute phases [40].

Normal liver and lung tissues exhibit clearly demarcated and persistent DNA damage patterns corresponding to MRT irradiation geometry (Figure 1A and Figure 3A). Tumour tissues, however, exhibit a less-defined damage pattern at early timepoints (Figure 4A). Macrophages closely associate with these regions of DNA damage, showing, for the first time, a distinct organization of macrophage populations in normal liver and lung tissue along the microbeam paths (Figure 1B and Figure 2B). To our knowledge, this specific accumulation of macrophages in areas of high dose deposition has not been described in previous studies of spatially fractionated RT. In the liver, this distinct pattern was present at 48 h and persisted up to 7 days post-irradiation, with a decline in density (Figure 1D). We were not, however, able to identify this type of specific organization of macrophages earlier than 7 days post-irradiation for normal lung tissue and lung carcinomas.

The sharp organization of F4/80+ macrophages densely populating the microbeam path in the liver are most likely resident liver macrophages, Kupffer cells, since recruited monocytes from the blood stream likely would not yet have differentiated into mature macrophages [40]. Nevertheless, this observation does not exclude the possibility of simultaneous immune cell recruitment, especially knowing that high peak-dose MRT provokes tumour vasculature disruption, creating a gate for the infiltration of different immune cells [41,42]. We indeed show a transient recruitment of CD11b+ myeloid progenitors which infiltrate the beam path by 48 h post-MRT in the liver (Figure 1C) and clear by 7 days (Figure 1D, right panel).

Macrophage populations of the liver consist of the resident Kupffer cells and bone-marrow derived monocytes, which migrate to the liver following injury [32]. Kupffer cells are strongly F4/80+ positive [27], account for the vast majority of hepatic macrophages [43] and are essential for repair following acute injury [44]. The type of injury, however, influences the inflammatory microenvironment, which in turn has a major impact on phenotypic changes of both resident and monocyte-derived macrophage populations, dictating their role in either resolving or aggravating tissue injury [45,46]. Typically, Kupffer cells are tolerogenic phagocytes, while bone marrow-derived populations are inflammatory [47]. It is apparent that resident macrophages of the liver respond to MRT-induced damage without eliciting a strong systemic immune response, as the recruitment of bone marrow-derived monocytes (Figure 1C,D, right panel) or peritoneal macrophages (Supplementary Figure S1) was minimal or absent, respectively. Interestingly, investigations into wound healing following focal sterile injury of the liver revealed a similar dynamic response by macrophages. Macrophages initially demarcated the site of injury within 48 h, where they then underwent a phenotypic conversion from a classical pro-inflammatory into non-classical macrophage populations that promoted tissue repair [48]. Therefore, the immune response to MRT-induced injury may not require the mobilization of monocytes from the bone marrow, as Kupffer cells may be sufficient scavengers of damaged cells induced by MRT, resulting in tissue-protective immunological tolerance and a non-inflammatory liver microenvironment [46].

Similarly, the lung also has resident macrophage populations residing in the alveolar and interstitial compartments [36], both expressing CD68 [49], with monocyte migration following injury contributing to inflammation and repair [50]. Thoracic irradiations have shown an increase in macrophage infiltration and pro-inflammatory activation in the lung [51]. Macrophages are responsible for the clearance of dying cells, and tissue-resident macrophages can efficiently clear large volumes of debris. When the injury is extensive, monocyte-derived alveolar macrophages support the onset of chronic inflammation [14] and subsequent fibrosis [52]. Since MRT only induces localized damage, it is, therefore, possible that resident macrophage populations of the normal liver and lung tissues can adequately clear the damaged cells confined to the microbeam path without the large and diffuse recruitment of potentially damaging inflammatory cells.

However, there is a different temporal dynamic in the mobilization of lung macrophages to the beam path with respect to that observed in the liver (7 days vs. 48 h). This may be explained by differences in tissue density, as the low cellular density of the lung may hinder the rapid migration of alveolar macrophages to the affected areas. On the other hand, this observed delay in the lung might also underlie a different origin of these macrophages, suggesting that the macrophages accumulating in the beam path of the lung may be a mix of resident and recruited cell populations. This tissue specificity emphasizes the need to study MRT immune responses in a tissue specific manner in order to accurately determine organ-specific responses. This characterization is particularly important since resident macrophage populations can differ in endogenous receptor expression, as depicted here, between the liver and lung: Kupffer cells demonstrate a high expression of F4/80, while alveolar macrophages have low F4/80 expression [27] and a high expression of CD68 [53].

The fact that MRT induces a localized and “controlled” immune response and does not trigger generalized and uncontrolled inflammation in the normal lung might be directly related to the absence of fibrosis 6 months post-treatment in mice [28] and even after 1 year in rats [54] when microbeams of 50 µm are applied. We expect that the liver would show similar responses with respect to the absence of long-term toxicity, in particular fibrosis, based on preliminary data collected by our laboratory not presented here.

The role of the macrophages following MRT may differ between normal and tumour tissues. Macrophages are heterogenous with pro- and anti-inflammatory polarization, with an anti-inflammatory phenotype associated with tumour progression (reviewed by [55]). While in normal tissues, we see the clear demarcation of the MRT beam path throughout the irradiated field, this localization is restricted to the periphery of the lung carcinoma samples. This may be explained by the presence of a necrotic core visible in the middle of the tumour. These necrotic areas are not vascularized and, as such, not supplied with oxygen and nutrients, and it is known that TAMs generally accumulate in pre-necrotic zones [56]. Although there are previous studies indicating a higher infiltration of TAMs in tumours after MRT compared to homogenous beam radiation [4,38], this specific immunostaining pattern has never been documented. Furthermore, tumour growth rates affect the maintenance of MRT geometry, with cellular migration disrupting the localization of peak- and valley-irradiated cells, making geometry demarcation at later timepoints post-irradiation difficult [57]. Therefore, this may account for the absence of macrophage organization into MRT-specific geometry in highly dynamic tumour tissue such as melanoma (Supplementary Figure S3B,C) compared to the LLC1 carcinoma (Figure 4 and Figure 5), which has a slower growth rate and more dense composition. This is also mirrored by the lack of a crossfire irradiation pattern with γH2AX in the lung carcinomas.

In the context of radiation, macrophage reprogramming is dependent on the dose and the type of radiation-induced cell death. The major factor in macrophage recruitment/activation following ionizing radiation depends on the type of signalling elicited by the tumour following exposure. This may depend on the type of radiation and the modality in which it is delivered. The production of cytokines and chemokines following ionizing radiation potentially varies with modality and may differentially influence macrophage polarization [22]. It has been shown that high doses (>10 Gy) of conventional irradiation increase M2-like, anti-inflammatory macrophage populations [58], while moderate doses (1–10 Gy) potentiate M1-like phenotypes [59]. The impact of radiation itself on macrophage function may be a key determinant of the tumour response to RT. Previous work has shown differential radiosensitivity between macrophage phenotypes with the M2, anti-inflammatory phenotype, having a greater degree of radiation resistance compared to the M1pheontype [60]. In contrast, during the acute phases following radiation, quiescent M1 macrophages were less sensitive to radiation-induced DNA damage and persisted over M2 populations [61]. Here, indeed, we show a mixed macrophage phenotype (M1- and M2-like) along the microbeam paths in the MRT-treated lung carcinoma. These regions, however, exceed the 50 µm width of the incident microbeam, indicating that the dose fall-off/gradient on either side of the peak evokes an immune response. This is in line with the difference in the macrophage phenotype previously described in response to low and high doses. The clear phagocytic component shown among these macrophages (Figure 5B) is also a typical response to radiation-induced injury [40]. We believe that this TAM concentration coincides with the area in which MRT induces vascular disruption. The presence of the M2-like TAMs might be a tentative neo-angiogenic response of the tumour to this phenomenon following MRT. The presence of cells expressing the CD206 receptor but not CD68 (pan-macrophage) may indicate a different cell type, most likely tumour-infiltrating dendritic cells [62]. Nevertheless, it must also be emphasized that the classical M1/M2 phenotypic distinction was primarily demonstrated by in vitro experimentation and that the recent advent of new technologies, first and foremost single cell RNA sequencing, showed that the macrophage response to injury in vivo is much more complex and is in continuous flux [63]. It is, therefore, too simplistic to separate them into two distinct categories. Only a future, full transcriptional characterization of these macrophages will shed light on their specific phenotypic activation in response to peak doses of hundreds of Gray.

In the context of spatially fractionated radiation therapy (SFRT), it has been shown that conventional-source SFRT is a powerful immune modulator [64]. Spatial fractionation may, therefore, have a dual effect on immunostimulatory responses, with high-dose deposition regions inducing immunogenic signalling, while at the same time preserving resident immune cells in the low-dose regions. Kanagavelu et al. [64] have shown that local ablative peak dose SFRT for LLC1 tumours induced increased systemic secretion of inflammatory cytokines, including IL-2, which is produced by macrophages and T-cells. MRT uses synchrotron-generated X-rays to deliver large doses in parallel microbeams and is therefore different to conventional RT due to its highly non-uniform dose distribution, ultra-high dose rates and kilovoltage rather than megavoltage photons. We have recently demonstrated that MRT delivered in therapeutic and sub-therapeutic doses evokes a long-lasting effect on the recruitment of macrophages to irradiated tumours and normal tissues [5,19]. Increased frequencies of activated macrophages have also been found in normal tissues outside the irradiated field attributable to abscopal signalling. Moreover, no cellular abscopal effects were observed in CCL2/MCP1 KO mice and wild-type mice injected with anti-CSF1R neutralizing antibody, which renders mice macrophage-depleted, indicating that macrophages and CCL2/MCP1, a cytokine that recruits macrophages to the site of an injury, play key roles in the generation and propagation of abscopal effects in normal tissues [65]. Forrester et al. [7] demonstrated an increase in TNF expression after MRT irradiation, further supporting the involvement of macrophages in both local tumour control and the systemic response to MRT irradiation, therefore presenting an attractive target to modulate these effects with a desirable outcome. However, when Yang et al. [6] irradiated EMT6.5 mouse mammary carcinomas with synchrotron MRT or conventional radiation, an increase in macrophage and neutrophil infiltration into irradiated tumours was observed only after conventional RT, but not MRT [6]. This contradicts our data and could be explained by the different tumour model used in their study, as well as the use of different MRT parameters (25 µm microbeam vs. 50 µm and in width and 200 µm microbeam spacing vs. 400 µm spacing in the current study). Nevertheless, this discrepancy underlies a necessity to further clarify the influence of such MRT parameters on the tissue immune responses to MRT.

Given the clinical relevance of macrophage populations as a prognostic indicator in cancer patients, future work quantifying these specific populations following MRT is needed in order to assess the impact of MRT-induced macrophage recruitment in treated tumours and their implication in treatment success and normal tissue sparing. Future experiments will clarify the function and nature of these macrophages that accumulate within the microbeam paths and whether other types of immune cell populations follow the same behaviour.

## 5. Conclusions

We have shown, for the first time, that MRT is able to induce targeted macrophage accumulation localized to the microbeam path. Utilizing MRT to induce targeted immune responses in tissues is of great interest not only for tumour control but also as a strategy to study macrophage behaviour in any given tissue with the precise delivery of controlled sterile injury. This may be of great interest for the fields of tissue regeneration and wound healing, opening up a new application for synchrotron-generated microbeams.

## Figures and Tables

**Table 1:** Summary of tissue samples analysed for the listed markers with corresponding irradiation parameters (microbeam width/microbeam spacing, peak dose), markers and time points.

Tissue Sample	MRT Parameters	Marker	Time Points
Liver	50/400 µm 400 Gy x2′ cross-fired	γH2AX, F4/80, CD11b, GATA-6	12, 24 h, 48 h
			7 days
Lung		γH2AX, CD68	12, 24, 48 h
			7 days
Ear	50/200 µm	γH2AX, CD68, CD11b	2, 7 days
	800 Gy		
Lung Carcinoma	50/400 µm	γH2AX, CD68, CD206, Dectin-1, Ly6C	3, 7, 16 days
	400 Gy x2′ cross-fired		
Melanoma	50/200 µm	γH2AX, C68, CD206	2 h
	400 Gy		2, 7 days

## Data Availability

The data presented in this study are available on request from the corresponding author.

## Supplementary Materials

The following are available online at https://www.mdpi.com/article/10.3390/biomedicines10040735/s1, Figure S1: Infiltrated macrophages in the liver are GATA-6 negative; Figure S2: Macrophages in non-irradiated lung carcinoma; Figure S3: Abundant DNA damage in melanomas after MRT and absence of macrophages localized infiltration along the microbeams.
