# Supplementary material for: Targeted Accumulation of Macrophages Induced by Microbeam Irradiation in a Tissue-Dependent Manner
Source: Biomedicines. 2022 Mar 22;10(4):735. doi: 10.3390/biomedicines10040735 (PMC9025837; doi:10.3390/biomedicines10040735)
Supplement: Supplementary file 1 [file biomedicines-10-00735-s001.zip › biomedicines-1620007-supplementary.pdf]

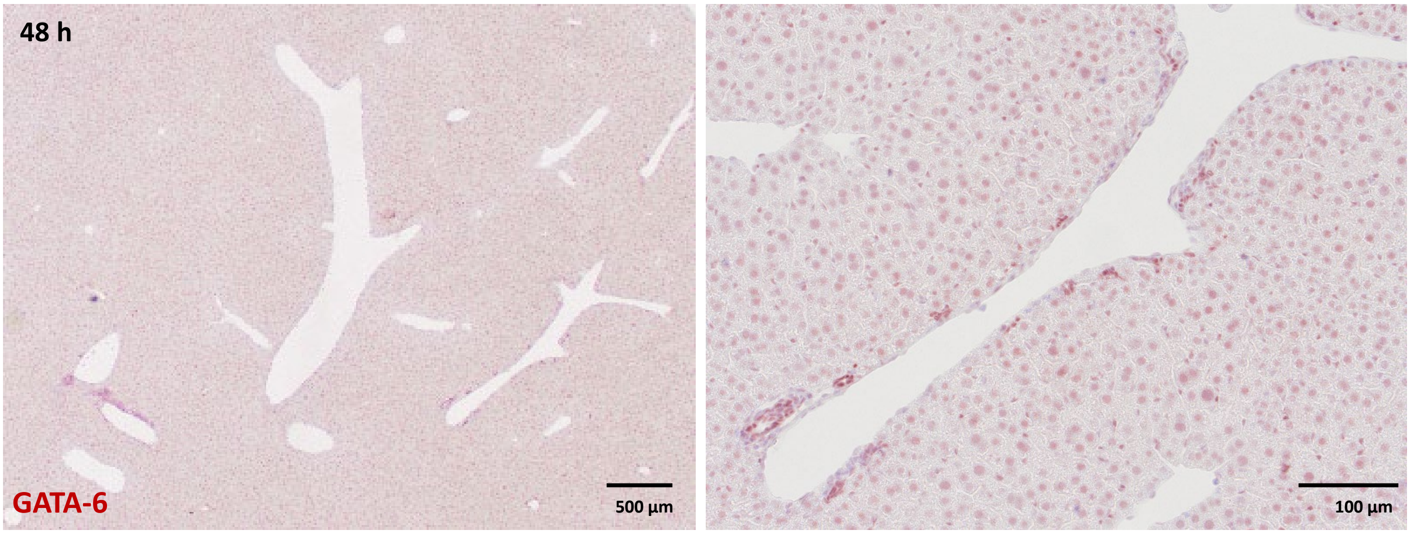

Figure Suppl. S1 – Infiltrated macrophages in the liver are GATA-6 negative. GATA-6 staining (NovaRED) reveal no patterned cellular distribution and stain a minor macrophage infiltrate around blood vessels and biliary ducts walls at 48 hours post-MRT.

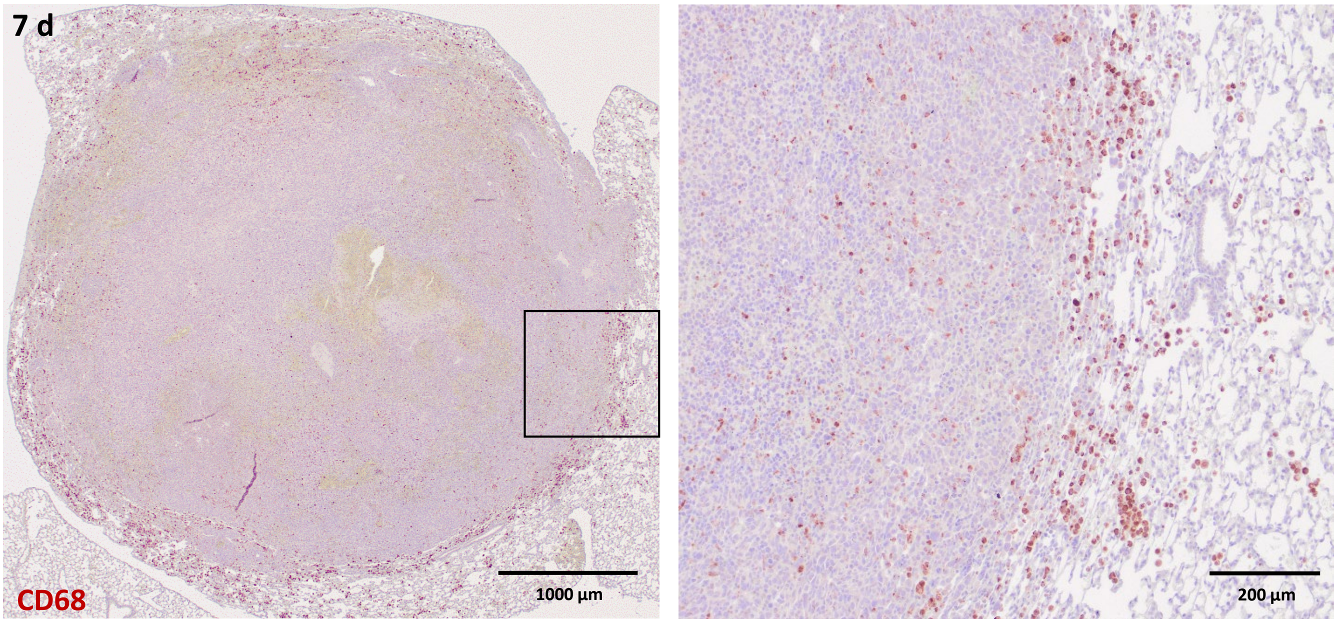

Figure Suppl. S2 – Macrophages in non-irradiated lung carcinoma. CD68 staining (NovaRED) in non-irradiated control LLC1 lung carcinoma. Macrophage population is restricted to the periphery of the tumour without patterned infiltration. Tumour is a representative non-irradiated control from the 7 day post-irradiation group. Left panel: overview of tumour showing the entire peripheral accumulation of macrophages. Right panel: zoom of indicated peripheral region showing no patterned infiltration.

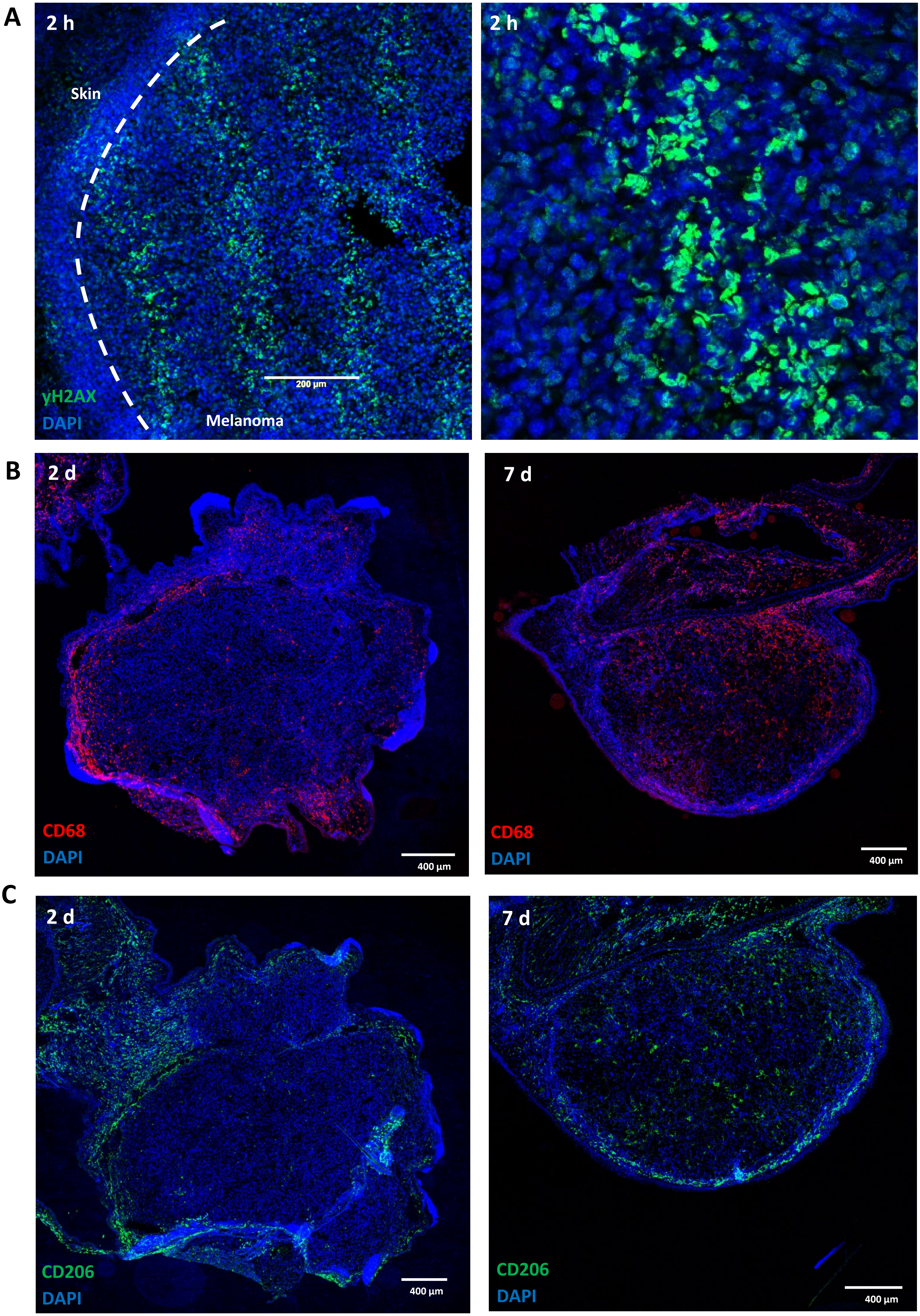
A
2 h
Skin
γH2AX
DAPI
200 µm
Melanoma
2 h
B
2 d
CD68
DAPI
400 µm
7 d
CD68
DAPI
400 µm
C
2 d
CD206
DAPI
400 µm
7 d
CD206
DAPI
400 µm

Figure Suppl. S3 – Abundant DNA damage in melanomas after MRT and absence of macrophages localized infiltration along the microbeams. Two hours post-MRT, harvested melanomas were stained with the anti-γH2AX antibody (green) and nuclear dye DAPI (blue). The white dashed line indicates the border between the skin (left side of the first image) and the melanoma (right side). Images at two different magnification are shown. **(B)** CD68 staining (red) and **(C)** CD206 staining (green) reveal no patterned infiltration of macrophages after 2 and 7 days post-MRT irradiations.
